# Maternal and Cord Blood Vitamin D Status and Anthropometric Measurements in Term Newborns at Birth

**DOI:** 10.3389/fendo.2018.00009

**Published:** 2018-01-25

**Authors:** Regina Wierzejska, Mirosław Jarosz, Magdalena Klemińska-Nowak, Marta Tomaszewska, Włodzimierz Sawicki, Michał Bachanek, Magdalena Siuba-Strzelińska

**Affiliations:** ^1^Department of Nutrition and Dietetics with Clinic of Metabolic Diseases and Gastroenterology, Institute of Food and Nutrition, Warsaw, Poland; ^2^Department of Neonatology, Mazovian Brodnowski Hospital, Warsaw, Poland; ^3^Clinic of Obstetrics, Gynecology and Oncology, 2nd Faculty of Medicine, Medical University of Warsaw, Warsaw, Poland

**Keywords:** vitamin D, blood, pregnancy, newborn, health

## Abstract

**Introduction:**

Vitamin D deficiency in pregnant women may result in reduced neonatal development due to the fact that systemic vitamin D status during fetal life depends on maternal concentrations. Some authors reported significant differences in neonatal anthropometric measurements depending on maternal vitamin D concentrations.

**Objective:**

The aim of this study is to evaluate the relationship between maternal and cord blood concentrations of vitamin D and neonatal anthropometric measurements at birth.

**Materials and methods:**

This study included 94 pregnant women, at term, who delivered at the Department of Obstetrics, Women’s Diseases and Gynecological Oncology, Medical University of Warsaw. Total serum 25(OH)D concentration was measured in mother–child pairs, and newborn anthropometric data were collected. A multiple regression analysis was used for statistical analysis.

**Results:**

No relationship between maternal and neonatal cord blood vitamin D concentrations vs. neonatal weight, length, head, and chest circumference at birth was found (*p* > 0.05). Severe vitamin D deficiency (<10 ng/ml) was detected in 10.6%, deficiency (10–20 ng/ml) in 39.4%, insufficiency (20–30 ng/ml) in 39.4%, and optimal vitamin D concentration (>30 ng/ml) only in 10.6% of the pregnant women. Cord blood vitamin D deficiency (<20 ng/ml) was found in 28.7% of the neonates.

**Conclusion:**

No differences between neonatal anthropometric measurements of infants born to mothers with normal and deficient vitamin D concentrations were found.

## Introduction

Vitamin D is responsible for a number of important functions in the fetus, and vitamin D blood saturation in the neonate is directly dependent on maternal levels. Vitamin D insufficiency in the mother results in neonatal insufficiency (1, 2), which may negatively affect the anthropometric parameters in the neonate, skeletal calcium score, the immune system, and increase the risk for asthma and type 1 diabetes in later life (3–5).

Currently, the recommended intake of vitamin D and its optimal concentration in the body are the source of much heated debate among medical researchers (6, 7). The American Academy of Pediatrics recommends that serum concentration of 25(OH)D in maternal blood be more than 32 ng/ml (8). In light of various reports, the general consensus is that it should exceed 30 ng/ml (9, 10). However, some experts are of the opinion that maternal levels of at least >20 ng/ml will ensure adequate vitamin D concentration in the neonate (11), while latest publications suggest that 25(OH)D concentration should be at least 40 ng/ml (2). With 30 ng/ml as the threshold value for adequate concentration, vitamin D insufficiency or deficiency is a common occurrence, affecting as many as 99–100% of pregnant women in Turkey (12, 13), 85% in India (14), 69–95% in Central Europe (15–17), 52–85% in Southern Europe (18, 19), 74% in the United States (20), and 63% in China (21). The contemporary lower reference range for cord blood vitamin D level is 20 ng/ml (1, 22). However, as in the case of maternal levels, there is no consensus regarding the optimal concentrations (23–26). The literature reports unanimously agree that cord blood serum concentration of vitamin D is directly correlated with maternal levels. Regardless, cord levels may be higher (24, 27–29), equal to (12, 14, 19), and lower than maternal venous vitamin D concentration (30, 31). Neonates born to mothers with adequate vitamin D concentrations during pregnancy are supplied until 8 weeks of life (2, 32). The contemporary literature questions whether the dose of vitamin D routinely administered to all newborns in the first weeks after birth should depend on the maternal concentrations and infant weight (3, 23).

The aim of this study is to evaluate the relationship between maternal and cord blood vitamin D concentrations and the anthropometric parameters of the newborn (weight, length, and head and chest circumference) as well as the Apgar score.

## Materials and Methods

### Study Design

The cross-sectional study was conducted among 100 pregnant women at the Department of Obstetrics, Gynecology and Oncology, Medical University of Warsaw. The study included women who delivered during two extreme seasons as far as exposure to sun is concerned (winter–summer) to investigate the widest range of vitamin D concentrations in the body. The winter group comprised women who delivered between December 2014 and February 2015, whereas the summer group included women who delivered between July and August 2015. This study included women who presented at the hospital on weekdays in the morning. The exclusion criteria were as follows: non-Polish nationality, multiple gestation, advanced stage of labor, chronic maternal diseases before pregnancy, and threatened course of labor. Informed written consent was obtained. Maternal blood was drawn after admission to the delivery ward (at the same time when blood was drawn for diagnostic purposes), and cord blood was drawn at delivery. Samples were immediately delivered to the hospital laboratory. Only term deliveries (94 patients: 45 from the winter group and 49 from the summer group) were included into the analysis. The Ethics Committee of the Institute of Food and Nutrition approved of the study (Code 10/162/KB/2014). Maternal and neonatal characteristics are presented in Table 1.

**Table 1:** Maternal and neonatal characteristics.

Number of women, *n* (%)	94 (100)
Winter group	45 (48.0)
Summer group	49 (52.0)
Age (years), mean ± SD	29.9 ± 4.3
Education, *n* (%)	
Higher	63 (67.0)
Other	31 (33.0)
Gravidity, *n* (%)	
Primiparas	40 (42.5)
Multiparas	54 (57.5)
Prepregnancy maternal body mass index, mean ± SD	22.9 ± 3.7
Weight gain during pregnancy, *n* (%)	
Low	23 (24.5)
Normal	31 (33.0)
Excessive	40 (42.5)
Gestational diabetes, *n* (%)	9 (9.5)
Smoking during pregnancy, *n* (%)	14 (15.0)
Professionally active during pregnancy, *n* (%)	54 (57.4)
Supplementation with vitamin/mineral preparations, *n* (%)	85 (90.4)
Supplementation with single-component vitamin D preparations, *n* (%)	13 (13.8)
Daily vitamin D consumption	
From diet (μg), median (range)	2.1 (0.2–11.5)
From supplements (μg), median (range)	12.0 (5.0–50.0)
Daily calcium consumption	
From milk and dairy products (mg), median (range)	596 (69–1872)
Dlaily caffeine consumption	
From coffee, tea, and energy drinks (mg), mean ± SD	67 ± 51
Sex of the newborn, *n* (%)	
Male	48 (51.0)
Female	46 (49.0)
Neonatal weight (g), mean ± SD	3515 ± 500
Neonatal length (cm), mean ± SD	55.4 ± 2.7
Apgar score (points), mean ± SD	9.9 ± 0.1
Neonatal head circumference (cm), mean ± SD	34.8 ± 1.4
Neonatal chest circumference (cm), mean ± SD	34.0 ± 1.9

### Laboratory Analysis and Data Collection

Total 25(OH)D [25(OH)D_2_ and 25(OH)D_3_] concentrations were measured in the blood using immunological tests (LIAISON^®^ 25 OH Vitamin D TOTAL Assay; DiaSorin). The lower detection threshold for vitamin D is 4.0 ng/ml. The intraassay and interassay CV were <8% and <11%, respectively. Neonatal data (sex, weight, length, Apgar score at 5 min, and head and chest circumference) were obtained from the hospital medical records. The anthropometric measurements were taken by the midwives immediately upon delivery. Weight was measured using a physician beam scale. The remaining measurements were taken with the use of a tape measure. The total neonatal length was measured from the vertex of the head to the soles (with the feet kept vertical at 90°). The occipital–frontal head circumference (tape was placed on the maximum protrusion of the occipital and supraorbital ridges) and the chest circumference (tape was placed horizontally on the sternum and lower tip of the shoulder blade) were measured. A Food Frequency Questionnaire, validated at the Institute of Food and Nutrition, was used to assess vitamin D, calcium, and caffeine consumption during the entire course of pregnancy. To precisely evaluate portion size, direct interviewing (face-to-face) and the “Photo Album of Meals and Products” were used for data collection. The questionnaire also included data on supplementation, patient lifestyle, and weight gain. As advised by the American Institute of Medicine, the recommended weight gain for pregnant women is 12.5–18 kg for underweight, 11.5–16 kg for normal weight, 7.0–11.5 kg for overweight, and 5–9 for obese women (33). These values were also used in our study. Lower or higher weight gain was considered as insufficient or excessive. The nutritional status was estimated using the body mass index (BMI). The content of vitamin D in vitamin/mineral supplements for pregnant women, as well as single-component vitamin D preparations, was estimated based on our earlier analysis (34). As many as 75% of the multicomponent supplements for pregnant women, which are available in Poland, contain a small amount of vitamin D (5–10 µg). Only single-component preparations contain larger amount of vitamin D (mean, 25 µg). The following criteria of maternal serum 25(OH)D concentrations were used: recommended level, >30 ng/ml; insufficiency, 20–30 ng/ml; deficiency, 10–20 ng/ml; and severe deficiency, <10 ng/ml (9, 27). As for cord blood, in accordance with the current recommendations of some experts, the concentration of ≥20 ng/ml was treated as the recommended level, whereas <12 ng/ml signified severe deficiency (1, 22).

### Statistical Analysis

A multiple regression analysis was used to investigate a possible relationship between selected baseline characteristics (serum vitamin D concentration, vitamin D and calcium consumption, gravidity, maternal age and education, prepregnancy BMI, weight gain during pregnancy, smoking, caffeine consumption, use of dietary supplements, professional activity during pregnancy, gestational diabetes, and sex of the neonate) and neonatal weight, length, and head and chest circumference. *p* < 0.05 was considered as statistically significant.

## Results

Overall, severe vitamin D deficiency (<10 ng/ml) was detected in 10 (10.6%), deficiency (10–20 ng/ml) in 37 (39.4%), insufficiency (>20–30 ng/ml) in 37 (39.4%), and optimal vitamin D concentration (>30 ng/ml) only in 10 (10.6%) pregnant women. The neonates had higher levels of vitamin D than the mothers (mean, 27.0 ± 11.1 ng/ml vs. 19.5 ± 7.8 ng/ml). As a consequence, vitamin D deficiency (<20 ng/ml) and severe deficiency (<12 ng/ml) were found in 27 (28.7%) and 9 (9.6%) cases, respectively.

No relationship between maternal and cord blood vitamin D concentrations and neonatal anthropometric measurements at birth (weight, length, and head and chest circumference) was found (*p* > 0.05). Due to the fact that mean Apgar score was close to the optimal values (9.9 points), that parameter was not included in the statistical analysis. Neontal somatic development was good in all neonates. No statistically significant differences were found between neonatal anthropometric parameters in the winter and summer groups although vitamin D concentrations were higher in the summer compared to the winter group [mean, 22.2 ± 6.5 ng/ml vs. 16.3 ± 8.0 ng/ml (*p* = 0.0003) for the mothers and 31.3 ± 9.4 ng/ml vs. 22.0 ± 11.0 ng/ml (*p* = 0.0001) for the neonates]. Weight gain during pregnancy was the only factor that proved to be associated with neonatal body weight and length. Children born to mothers whose weight gain in pregnancy was lower than the recommendations were 302 g lighter (*p* = 0.0405) and 2.4 cm shorter (*p* = 0.0025), compared to infants born to mothers with normal weight gain (Tables 2 and 3).

**Table 2:** Relationship between selected parameters and neonatal weight.

	Regression beta coefficient (SE)	95% confidence interval	*p*-Value
Maternal serum vitamin D concentration	−5.84 (15.491)	−36.68 to 25.00	0.7070
Cord blood vitamin D concentration	−0.05 (11.230)	−22.41 to 22.31	0.9965
Vitamin D consumption	1.72 (2.951)	−4.15 to 7.59	0.5616
Calcium consumption	5.90 (12.151)	−18.29 to 30.09	0.6284
Supplementation with vitamin/mineral preparations	−232.65 (181.872)	−594.73 to 129.43	0.2046
Pre-pregnancy body mass index:			
underweight	15.69 (199.141)	−380.77 to 412.15	0.9374
overweight/obesity	70.77 (151.218)	−230.28 to 371.82	0.6411
Low weight gain vs. normal gain	−301.77 (144.827)	−590.10 to −13.44	0.0405
Excessive weight gain vs. normal gain	55.03 (137.407)	−218.52 to 328.59	0.6899
Gestational diabetes	120.94 (176.572)	−230.59 to 472.47	0.4954
Smoking	57.71 (159.212)	−259.26 to 374.67	0.7180
Caffeine consumption	−154.11 (138.210)	−429.27 to 121.04	0.2683
Age	10.91 (13.953)	−16.87 to 38.69	0.4365
Education	−144.76 (125.909)	−395.43 to 105.91	0.2538
Gravidity	−57.38 (116.065)	−288.44 to 173.69	0.6225
Professional activity during pregnancy	−60.90 (108.664)	−277.24 to 155.43	0.5768
Neonatal sex	−28.27 (110.419)	−248.10 to 191.56	0.7986

**Table 3:** Relationship between selected parameters and neonatal length.

	Regression beta coefficient (SE)	95% confidence interval	*p*-Value
Maternal serum vitamin D concentration	−0.00 (0.839)	−0.17 to 0.16	0.9765
Cord blood vitamin D concentration	0.02 (0.061)	−0.10 to 0.14	0.7747
Vitamin D consumption	−0.01 (0.016)	−0.04 to 0.02	0.5247
Calcium consumption	−0.04 (0.066)	−0.17 to 0.10	0.5936
Supplementation with vitamin/mineral preparations	−0.99 (0.985)	−2.95 to 0.97	0.3159
Pre-pregnancy body mass index:			
underweight	−0.24 (1.078)	−2.39 to 1.91	0.8247
overweight/obesity	−0.09 (0.82)	−1.72 to 1.55	0.9176
Low weight gain vs. normal gain	−2.44 (0.784)	−4.01 to −0.88	0.0025
Excessive weight gain vs. normal gain	−0.29 (0.744)	−1.77 to 1.19	0.6970
Gestational diabetes	0.60 (0.956)	−1.30 to 2.51	0.5295
Smoking	−0.14 (0.862)	−1.85 to 1.58	0.8758
Caffeine consumption	−0.93 (0.748)	−2.42 to 0.56	0.2189
Age	−0.03 (0.076)	−0.18 to 0.12	0.6666
Education	−0.68 (0.682)	−2.04 to 0.67	0.3199
Gravidity	0.02 (0.629)	−1.23 to 1.27	0.9740
Professional activity during pregnancy	0.26 (0.588)	−0.91 to 1.44	0.6548
Neonatal sex	−0.07 (0.598)	−1.26 to 1.12	0.9122

## Discussion

In our study, we found no association between vitamin D status and the somatic development of the newborns. Despite the fact that maternal vitamin D levels were diverse (minimum, 4 ng/ml; maximum, 37.7 ng/ml), no relationship between any of the investigated anthropometric parameters and maternal values was found. Also, regardless of the fact that predelivery maternal concentrations of vitamin D were too low in approximately 90% of the women, the overall condition of the neonates was good, even in cases classified as “severely deficient in vitamin D” (<10 ng/ml in maternal and <12 ng/ml in cord blood). Lack of a relationship between maternal vitamin D concentrations and neonatal measurements in Poland was reported by Skowrońska-Jóźwiak et al., although their study focused on insufficiently low vitamin D concentrations, which were confirmed in almost 69% of mothers of term infants (16). As for data from other countries, Rodriguez et al. and Eggemoen et al. found no connection between neonatal anthropometric parameters and maternal vitamin D levels, even despite the fact that vitamin D deficiency (<20 ng/ml) was detected in 19.7% and 51% of the women, respectively (18, 35). Loudyi et al. reported no association between maternal vitamin D status and neonatal weight at birth among the investigated women (vitamin D concentration ≤20 ng/ml in 90% of the cases) (36). Shakiba and Iranmanesh and Josefson et al. found no relationship between cord vitamin D concentration and neonatal weight and length (25, 31), while Dalgård et al. reported the same lack of relationship for neonatal weight and head circumference (37).

Nevertheless, some authors have postulated the existence of such a relationship and reported significant differences in neonatal size depending on vitamin D concentrations. An Indian study has demonstrated lower weight (by 480 g), length (by 9.5 cm), and head and chest circumference (by 4.5 and 4.8 cm, respectively) in neonates born to mothers with vitamin D deficiency compared to the recommended levels (14). Nobles et al. found a smaller difference (by 176 g) in the weight of infants born to mothers with vitamin D deficiency in the United States (20). As for cord blood, yet another study reported decreased neonatal length (by 0.5 cm) in a group of children with very low vitamin D concentrations (<4.8 ng/ml) compared to the recommended levels (37). Interesting results were reported by Lykkedegn et al., who demonstrated a U-shaped association between neonatal weight at birth and cord blood vitamin D concentrations. A significant weight gain was observed after the concentration values exceed 24 ng/ml (38).

In light of the conflicting data from individual studies, meta-analyses seem to be the most valuable sources of information. A meta-analysis of observational studies has indicated that lowered maternal vitamin D concentration (<15 ng/ml) results in lower neonatal weight at birth (by 131 g), but has no impact on the length and head circumference (6). Another meta-analysis has concluded that maternal vitamin D concentration of <20 ng/ml increases the risk for small-for-gestational age infant (39).

The conclusions on the effects of vitamin D supplementation during pregnancy are also conflicting. A meta-analysis of randomized clinical trials has found that vitamin D supplementation during pregnancy increases neonatal weight and length but does not affect the course of pregnancy. However, these authors emphasize the need for further studies to obtain more conclusive results (40). Harvey et al., in a systematic review, claimed that the available literature reports are not sufficient to confirm the existence of a relation between vitamin D concentration and neonatal condition at birth, or even to recommend obligatory vitamin D supplementation in pregnant women (7).

The results of our study did not reveal deteriorated neonatal condition at birth among children born to mothers with low vitamin D levels compared to optimal concentrations. Nevertheless, several limitations might have biased the final results, especially the fact that the analysis of vitamin D level was a single test and was conducted on the day of the delivery, which does not signify that the concentration was typical for the entire course of pregnancy or at least its significant part. The second limitation of our study was a relatively small sample size, which was the result of the number of deliveries at the Clinic and the nature of the study, i.e., the winter–summer group, so caution is advised when formulating final conclusions.

## Conclusion

Our study did not demonstrate a relationship between the anthropometric features of the neonates and maternal and cord blood vitamin D levels. In the absence of differences in neonatal condition between infants born to mothers with vitamin D deficiency and with adequate levels, it seems safe to conclude that the currently recommended vitamin D levels in pregnant women (>30 ng/ml) do not need to be reached to ensure proper fetal growth. Regardless, vitamin D performs various essential functions in the human body, some of which remain to be fully elucidated, so its concentration during pregnancy should not only be perceived through neonatal anthropometric measurements but also be perceived through long-term development and health condition of the children.

## Ethics Statement

This study was carried out in accordance with the recommendations of the Ethics Committee of the Institute of Food and Nutrition approved of the study (Code 10/162/KB/2014) with written informed consent from all subjects. All subjects gave written informed consent in accordance with the Declaration of Helsinki. The protocol was approved by the Ethics Committee of the Institute of Food and Nutrition.

## Author Contributions

RW conceived the idea for the study. RW, MJ, and WS contributed to the design of the research. RW, MK-N, MT, MB, and MS-S collected the data. RW and MK-N analyzed the data and wrote the paper. All authors edited and approved the final version of the manuscript.

## Conflict of Interest Statement

The authors declare that the research was conducted in the absence of any commercial or financial relationships that could be construed as a potential conflict of interest. The reviewer JK and handling editor declared their shared affiliation.
